# Paraphyletic genus *Ditylenchus* Filipjev (Nematoda, Tylenchida), corresponding to the *D. triformis*-group and the *D. dipsaci*-group scheme

**DOI:** 10.3897/zookeys.568.5965

**Published:** 2016-02-23

**Authors:** Yuejing Qiao, Qing Yu, Ahmed Badiss, Mohsin A. Zaidi, Ekaterina Ponomareva, Yuegao Hu, Weimin Ye

**Affiliations:** 1 China Agriculture University, Beijing, China; 2 Eastern Cereal and Oilseed Research Center, Agriculture and Agri-Food Canada, Ottawa, Ontario, Canada; 3 Nematode Assay Section, Agronomic Division, North Carolina Department of Agriculture & Consumer Services, NC, USA

**Keywords:** *Ditylenchus*, ITS, 18S ribosomal DNA, RAPD, genetic variations, mycophagous, plant parasitic nematodes, phylogeny

## Abstract

The genus *Ditylenchus* has been divided into 2 groups: the *Ditylenchus triformis*-group, and the *Ditylenchus dipsaci*-group based on morphological and biological characters. A total of 18 populations belong to 5 species of *Ditylenchus* was studied: *Ditylenchus africanus*, *Ditylenchus destructor*, *Ditylenchus myceliophagus* and *dipsaci*, *Ditylenchus weischeri*, the first 3 belong to the *Ditylenchus triformis*-group, the last 2 the *Ditylenchus dipsaci*-group. The species of *Ditylenchus triformis*-group were cultured on fungi, while the species from *Ditylenchus dispaci*-group cultured on excised roots of plant hosts in petri dish. DNA sequences of regions of the nuclear ribosomal first internal transcribed spacer (ITS1) and the small subunit 18S were PCR amplified, sequenced and the phylogenetic analyses also including the sequences of the closely related species from the GenBank. The randomly amplified polymorphisms of genomic DNA (RAPD) were also generated. Two clusters or clades corresponding to the 2 groups were consistently observed with significant statistical support from the 3 datasets. The phylogenetic analysis also revealed that the genus is paraphyletic, separating the 2 groups by species of *Anguina* and *Subanguina*.

first internal transcribed spacer

## Introduction

The genus *Ditylenchus* Filipjev (1936) consists of 80-90 accepted species (Brzeski 1991) of either mycophagous, entomophlic or plant parasitic species. The genus includes some of the most destructive nematode pests, e.g. the mushroom spawn nematode *Ditylenchus myceliophagus* Goodey 1958, the potato rot nematode *Ditylenchus destructor* Thorne 1945, and the stem and bulb nematode *Ditylenchus dipsaci* (Kühn, 1857) Filipjev 1936, the latter two are also internationally quarantined. As the climate change intensifies and international trade increases, invasive alien species such as nematode species are increasingly becoming serious problems, as demonstrated by the recent outbreak of the stem and bulb nematode in central Canada and the neighboring states of USA, (Yu et al. 2010, Qiao et al. 2013), and the recent finding of potato rot nematode in Ontario (Yu et al. 2012), which was the first finding on the continental Canada for the pest.

Taxonomy of the genus both above and below the rank has been confusing. The genus was first placed in the family Tylenchidae of Tylenchina (Filipjev 1936), moved to *Anguillulina* Schneider (1939) and moved again to Anguinidae (Paramonov 1970). The family has been moved between Hexatylina and Tylenchina (Siddiqi 1986, and 2000). Within the genus, species delimitation based on morphology has been rather arbitrary, since many morphometrical characters are highly variable and only a few were constant enough to be used for taxonomic purposes (Fortuner 1982). The species complex of *Ditylenchus dipsaci* (Sturhan & Brzeski, 1991) makes this situation even more confusing. Recently applications of molecular methods have provided new tools for researchers to better understand the biology and taxonomy of the genus. For example, *Ditylenchus weischeri* Chizhov, Borisov & Subbotin (2010) has been separated as a valid species from the *Ditylenchus dipsaci* species complex, *Ditylenchus gigas* Vovlas (2011) from the giant race of *Ditylenchus dipsaci*, and *Ditylenchus africanus* Wendt (1995) from *Ditylenchus destructor*. Recent phylogenetic studies of ribosomal DNA indicated that the genus may be paraphyletic (Holterman et al. 2009; Giblin-Davis et al. 2010).

Two groups of the genus were recognized: the *Ditylenchus triformis*-group and *Ditylenchus dipsaci*-group (Siddiqi 1980). The *Ditylenchus triformis*-group includes species with a rounded tail tip, lateral fields of six lines, and having mycophagous life cycle such as *Ditylenchus destructor* and *Ditylenchus myceliophagous*, while the *Ditylenchus dipsaci*-group includes obligate plant parasites with a sharp-pointed tail tip and lateral fields of four lines. Those entomophlic species such as *Ditylenchus halictus* are also mycophagous; belong to the *Ditylenchus triformis*-group (Giblin-Davis et al. 2010).

The objective of the study was to use three molecular datasets, namely ITS1 and 18S fragment sequences of ribosomal DNA and RAPD polymorphisms of genomic DNA, to determine the phylogenetic relationships of the two groups of *Ditylenchus* species.

## Material and methods

### Nematode population

Live nematodes of eight populations of *Ditylenchus destructor*, six populations of *Ditylenchus dipsaci*, one of each *Ditylenchus africanus*, *Ditylenchus weischeri* and *Ditylenchus myceliophagus* from different regions of three countries were collected (Table 1). Species identifications were confirmed using morphological and molecular methods.

**Table 1.** Origins, hosts and access numbers of *Ditylenchus* species and populations used in this study

Code	Species	Location	Host	Accession No.	
				ITS	18S
**CH01**	*Ditylenchus destructor*	Inner Mongolia, China	Sweet potato	KJ567140	KJ492926
**CH02**	*Ditylenchus destructor*	Jilin, China	Sweet potato	KJ567141	KJ492927
**CH03**	*Ditylenchus destructor*	Henan, China	Sweet potato	KJ567142	KJ492928
**CH04**	*Ditylenchus destructor*	Shandong, China	Sweet potato	KJ567143	KJ492929
**CH05**	*Ditylenchus destructor*	Jiangsu, China	Sweet potato	KJ567144	KJ492930
**CH06**	*Ditylenchus destructor*	Hebei, China	Sweet potato	KJ567145	KJ492931
**CA01**	*Ditylenchus destructor*	Ontario, Canada	Sweet potato	KJ567146	KJ492932
**CU01**	*Ditylenchus destructor*	Clemson University, USA	Sweet potato	KJ567147	KJ492933
**CA02**	*Ditylenchus dipsaci*	Ontario, Canada	Onion	KJ567148	KJ492934
**CU02**	*Ditylenchus dipsaci*	Clemson University, USA	Garlic	KJ567149	KJ492935
**CA03**	*Ditylenchus dipsaci*	Ontario, Canada	Garlic	KJ567150	KJ492936
**CA04**	*Ditylenchus dipsaci*	Ontario, Canada	Garlic	KJ567151	KJ492937
**CA05**	*Ditylenchus dipsaci*	Ontario, Canada	Garlic	KJ567152	KJ492938
**CA06**	*Ditylenchus dipsaci*	Ontario, Canada	Garlic	KJ567153	KJ492939
**DA**	*Ditylenchus africanus*	South Africa	Peanut	KJ567154	KJ492940
**DW**	*Ditylenchus weischeri*	Manitoba, Canada	Canada thistle	KJ567155	KJ492941
**DM**	*Ditylenchus myceliophagus*	Ontario, Canada	Grass	KJ567156	KJ492942

### Nematode culturing

*Ditylenchus destructor*, *Ditylenchus myceliophagus* and *Ditylenchus africanus* were cultured on *Fusarium oxysporium* on 10% potato dextrose agar (PDA). *Ditylenchus dipsaci* and *Ditylenchus weischeri* were cultured on yellow pea and soybean excised roots on White’s medium (White 1939) respectively but attempts were also made to culture *Ditylenchus dipsaic*, and *Ditylenchus weischeri* on *Fusarium oxysporium*.

### Sample preparation

PDA with fungus media and roots infested with nematodes were cut into small pieces and nematodes extracted using the Baermann funnel method (Baermann 1917).

### DNA extraction

One or two extracted nematodes were subjected to DNA extraction. The nematodes were crushed in microtubes containing 40 μL 10×PCR buffer (100 mM Tris-HCl, pH 9.0 at 25 °C, 500 mM KCl, 15 mM MgCl_2_), 10 μL Proteinase K (1 mg/mL), 50 μL distilled water. The microtubes were incubated for 1.5 h at 65°C followed by 15 min at 95 °C and stored at -20 °C. DNA templates were quantified using a NanoDrop ND-1000 Spectrophotometer (Wilmington, DE, USA).

### Sequencing and alignment of ITS1 and 18S regions of nuclear rRNA

A region of the internal transcribed spacer 1 (ITS1) gene was amplified using the primers ITS-F (5’-TTGATTACGTCCCTGCCCTTT-3’), ITS-R (5’-ACGAGCCGAGTGATCCACCG-3’). The amplification protocol was: initial denaturation at 94 °C for 3 min, followed by 40 cycles of denaturation (30 s at 94 °C), annealing (45 s at 58 °C), and extension (2 min at 72 °C), with a final extension for 10 min at 72 °C. A region of the small subunit (SSU) 18S rRNA gene (18S) was amplified using the primers 18S-F (5’-TTGGATAACTGTGGTTTAACTAG-3’) and 18S-R (5’-ATTTCACCTCTCACGCAACA-3’). The amplification condition was: 95 °C for 3 min, followed by 40 cycles of 30 s at 95 °C, 45 s at 60 °C and 2 min at 72 °C, with final extension of 10 min at 72 °C. All PCR reactions were performed in 25 ul volumes including 10 ng DNA, 2.5 µl 10×PCR buffer, 1.5 µl 2.5 mM dNTPs, 0.2 ul 10 µM primers and 0.25 µl Titanium Taq DNA polymerase (supplier). The ITS and 18S fragments were sequenced in-house with an ABI Prism 377 sequencer (Perkin Elmer) in both directions and unambiguous consensus sequences obtained. The sequences were deposited into the genBank database. DNA sequences were aligned by Clustal W (http://workbench.sdsc.edu, Bioinformatics and Computational Biology group, Dept. Bioengineering, UC San Diego, CA). The sequences were compared with those of the other nematode species available at the genBank sequence database using the BLAST homology search program. The model of base substitution was evaluated using MODELTEST (Posada and Crandall 1998; Huelsenbeck and Ronquist 2001). The Akaike-supported model, the base frequencies, the proportion of invariable sites and the gamma distribution shape parameters and substitution rates were used in phylogenetic analyses. Bayesian analysis was performed to confirm the tree topology for each gene separately using MrBayes 3.1.0 (Huelsenbeck and Ronquist 2001) running the chain for 1 × 106 generations and setting the “burnin” at 1,000. We used the Markov Chain Monte Carlo (MCMC) method within a Bayesian framework to estimate the posterior probabilities of the phylogenetic trees (Larget and Simon 1999) using 50% majority rule.

### RAPD (randomly amplified polymorphic DNA) and data analysis

Twenty seven random primers were used for RAPD analysis. These primers were previously shown to be suitable for inter-species comparison of *Ditylenchus* (Digby and Kempton 1987; Zouhar et al. 2007). All PCR reactions were performed in 25 µl volumes consisting of 1 µL of genomic DNA prepared earlier as described above, 2.5 µl of 10×PCR buffer, 1.25 µl of 2.5 mM dNTPs, and 0.25 µl of Titanium Taq DNA polymerase (Clontech Lab Inc.). Amplification conditions were as follows: an initial denaturation at 94 °C for 1 min, followed by 40 cycles of denaturation at 94 °C for 1min, annealing/extension at 72 °C for 1min and a final extension at 72 °C for 10 min. The PCR products were separated by electrophoresis (100V, 1h) in 2.0% agarose gels in TAE buffer with 180-200 ng DNA. The gels were stained with ethidium bromide, visualized and photographed under UV-light (Bio-rad DX, USA). All reactions were repeated twice for clear and stable banding patterns. The presence or absence of DNA fragments was scored as one or zero, respectively, in the binary matrix. Simple matching coefficients (SM) (Digby and Kempton 1987) and hierarchical cluster analysis were performed with NTSYS2.1 (Exeter Software, Setauket, NY). Cluster analysis, by the un-weighted pair method with arithmetic mean (UPGMA), was performed with the SAHN (sequential, agglomerative, hierarchical and nested clustering method). The robustness of the dendrogram was tested with 1000 bootstrap replicates using PAUP software (Swofford 2003).

## Results

*DNA sequences*: Ribosomal DNA fragments of the internal transcribed spacer 1 (404 bp) and fragments of the 18S ribosomal RNA gene (902 bp) were amplified and sequenced and sequences deposited in GenBank (www.ncbi.nlm.nih.gov/genbank). GenBank accession numbers are listed in Table 1.

**Figure 1.**
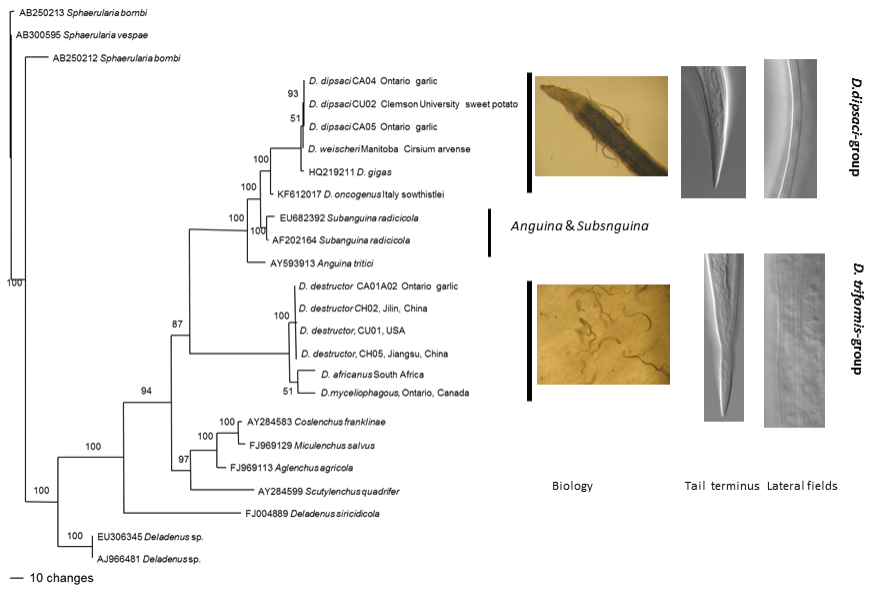
The 10001st Bayesian likelihood tree inferred from ITS sequences under GTR+I+G model (lnL = 9697.1895; freqA = 0.2646; freqC = 0.2062; freqG = 0.2602; freqT = 0.269; R(a) = 0.9399; R(b) = 3.4936; R(c) = 2.4954; R(d) = 0.5528; R(e) = 5.2698; R(f) = 1; Pinva = 0.4389; Shape = 0.7862). Posterior probability values exceeding 50% are given on appropriate clades.

**Figure 2.**
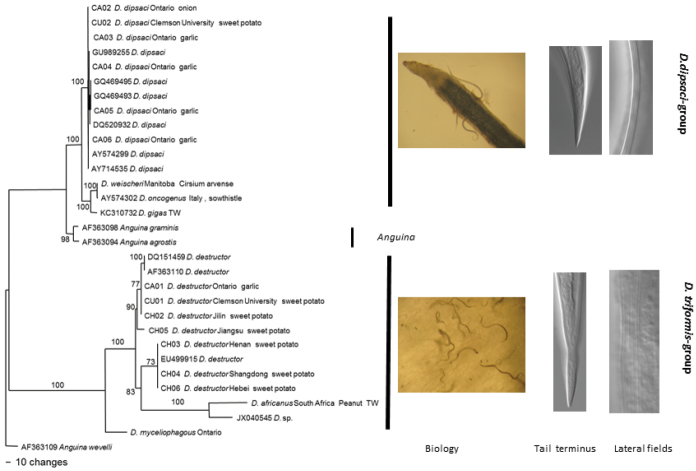
The 10001st Bayesian likelihood tree inferred from 18S sequences under GTR+I+G model (lnL = 9697.1895; freqA = 0.2646; freqC = 0.2062; freqG = 0.2602; freqT = 0.269; R(a) = 0.9399; R(b) = 3.4936; R(c) = 2.4954; R(d) = 0.5528; R(e) = 5.2698; R(f) = 1; Pinva = 0.4389; Shape = 0.7862). Posterior probability values exceeding 50% are given on appropriate clades.

*Phylogeny*: Phylogenetic trees based on the ITS1 and 18S sequences of rDNA are shown in Figures 1 and 2 respectively. The results are consistent for both ITS and 18S with species separating into two clusters, one cluster comprising *Ditylenchus destructor*, *Ditylenchus africanus* and *Ditylenchus myceliophagus*, and the second comprising *Ditylenchus dipsaci*, *Ditylenchus weischeri* and *Ditylenchus gigas*, with the groupings corresponding well with the tail endings. The 2 clusters were separated by species of *Anguina*.

**Figure 3.**
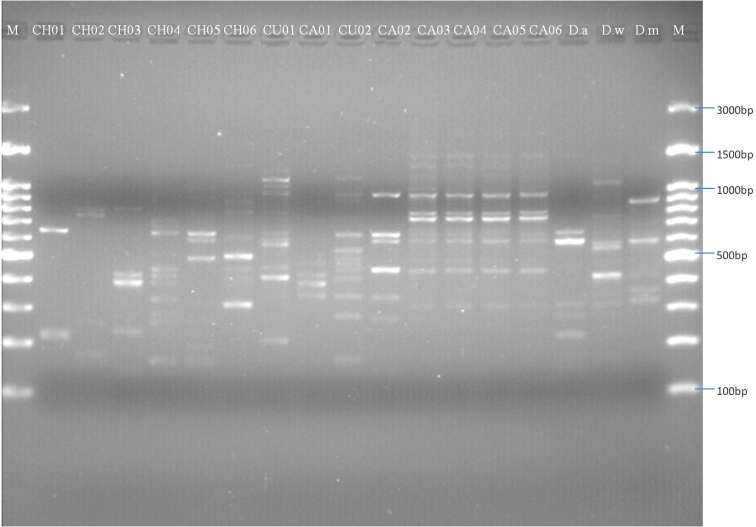
Random Amplified Polymorphic DNA (RAPD) profiles of all *Ditylenchus* species using primer OPG-05.

*RAPD analysis*: Among the 27 primers (excepting RAPD2, RAPD3, RAPD5, RAPD7, OPA17 and OPB16 which amplified no visible bands) 21 random primers produced clear and reproducible bands. A total of 212 bands ranging from 100-2000 bp in size were produced by the 21 primers. 121 and 42 polymorphic bands were obtained for *Ditylenchus destructor* and *Ditylenchus dipsaci* respectively, which suggests higher genetic variation among populations of the *Ditylenchus destructor* than those of *Ditylenchus dipsaci*. Figure 3 presents the RAPD profiles obtained from primers OPG-05 to exemplify the banding patterns observed.

**Figure 4.**
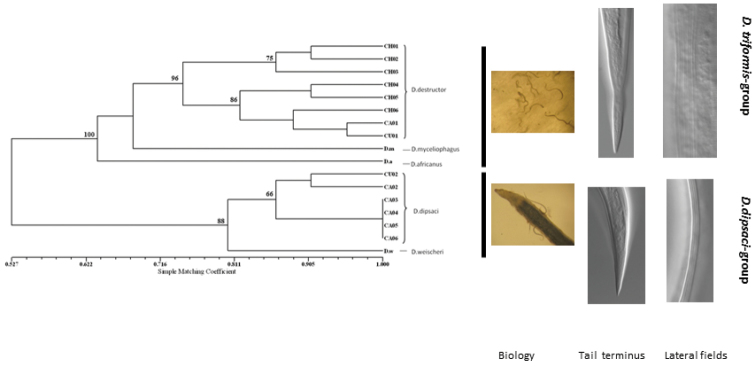
Un-weighted Pair Group Method with Arithmetic Mean (UPGMA) tree showing estimated average genetic distances among all *Ditylenchus* species based on simple matching coefficient obtained from RAPD analysis.

The RAPD binary data matrix and resulting simple matching coefficient (SM) are presented in Table 2. Figure 4 shows the dendrogram indicating the relationships among all collections. Species of *Ditylenchus* separated into two clusters consistent with the phylogenetic results based on the ITS1 and 18S sequences. *Ditylenchus destructor*, *Ditylenchus africanus*, and *Ditylenchus myceliophagus* comprised one cluster and *Ditylenchus dipsaci* and *Ditylenchus weischeri* the second cluster. All *Ditylenchus destructor* populations were in one cluster with similarity of 74.2%, and all six populations of *Ditylenchus dipsaci* in the other cluster with a higher degree of genetic similarity (87%).

**Table 2.** Similarity matrix (Simple Matching Coefficient) among all *Ditylenchus* species obtained with 21 primers and based on shared DNA fragments.

	CH01	CH02	CH03	CH04	CH05	CH06	CA01	CU01	CA02	CU02	CA03	CA04	CA05	CA06	DA	DW	DM
**CH01**	1.000																
**CH02**	0.909	1.000															
**CH03**	0.909	0.818	1.000														
**CH04**	0.681	0.681	0.681	1.000													
**CH05**	0.773	0.773	0.773	0.909	1.000												
**CH06**	0.773	0.773	0.773	0.909	0.818	1.000											
**CA01**	0.727	0.727	0.727	0.772	0.773	0.864	1.000										
**CU01**	0.773	0.773	0.773	0.818	0.818	0.909	0.955	1.000									
**CA02**	0.409	0.409	0.409	0.455	0.455	0.455	0.500	0.455	1.000								
**CU02**	0.682	0.591	0.682	0.455	0.455	0.455	0.591	0.545	0.909	1.000							
**CA03**	0.500	0.500	0.500	0.545	0.545	0.545	0.591	0.545	0.909	0.818	1.000						
**CA04**	0.500	0.500	0.500	0.545	0.545	0.545	0.591	0.545	0.909	0.818	1.000	1.000					
**CA05**	0.500	0.500	0.500	0.545	0.545	0.545	0.591	0.545	0.909	0.818	1.000	1.000	1.000				
**CA06**	0.500	0.500	0.500	0.545	0.545	0.545	0.591	0.545	0.909	0.818	1.000	1.000	1.000	1.000			
**DA**	0.591	0.591	0.591	0.727	0.727	0.636	0.591	0.636	0.545	0.545	0.636	0.636	0.636	0.636	1.000		
**DW**	0.591	0.500	0.591	0.455	0.545	0.455	0.500	0.455	0.818	0.727	0.818	0.818	0.818	0.818	0.545	1.000	
**DM**	0.591	0.591	0.591	0.727	0.727	0.727	0.773	0.727	0.636	0.727	0.636	0.636	0.636	0.636	0.636	0.545	1.000

## Conclusions

All three molecular data supports morphological schemes for this genus to be divided into two groups: *Ditylenchus triformis*-group and *Ditylenchus dipsaci*-group, and that the genus is paraphyletic dividing along the group line by *Anguina* and *Subanguina*.

## Discussion

The results of the study provide strong evidence for divide the genus into 2 groups, one for *Ditylenchus triformis*-group and *Ditylenchus dipsaci*-group, and genus is paraphyletic. Paraphyletic and polyphyletic taxa are nothing new to biosystematics, even in nematoda several taxa have been found either paraphyletic or polyphyletic: such as *Hoplolaimus* is paraphyletic (Bae et al. 2008, Ma et al. 2011) and Aphelenchoididae polyphyletic (Kanzaki et al. 2009). It is debateable whether non-monophyletic taxa should be accepted. However as taxonomy advances from traditional to phylogenetic; however, more and more researchers would reject paraphyletic or polyphyletic taxa since they are inconsistent with evolution.

When the genus *Ditylenchus* was established by Filipjev (1936) by synonymizing *Tylenchus dipasci* to *Ditylenchus dipsaci* it was placed in the family Tylenchidae (Nematoda: Tylenchida) as the sister genus to *Tylenchus*. Even today differences between species of the two genera are primarily morphometric, although now the genus is placed in the family of Anguinidae. There is some molecular evidence suggesting that one of the evolutionary paths of plant parasitism in nematodes is from algae-feeding nematodes *Tylenchus* to *Ditylenchus* (Holterman et al. 2009), which may be true for the obligate plant parasitic *Ditylenchus* species since the sharp-pointed tail tip is a feature in common for the two genera. Morphologically, the *Ditylenchus triformis*-group is closely related with *Safianema*, and there is also molecular evidence (Giblin-Davis 2010) that they belong to one clade, that the species of *Ditylenchus triformis*-group should be synonymized into *Safinema*, and there are also molecular evidences that *Safianema* and *Ditylenchus triformis*-group are closely related to Neotylenchidae (suborder Hexatylina) than to Tylenchidae (suborder: Tylenchina) (Robin-Davis 2010), and a rounded tail tip (shared characteristic for both *Ditylenchus triformis*-group and *Safianema*) and is a shared character in Hexatylina. To resolve the synonymization and the eventual high rank placement of the putatively synonymized *Safinema*, more studies are needed.
